# Effect of Dietary Counseling on a Comprehensive Metabolic Profile from Childhood to Adulthood

**DOI:** 10.1016/j.jpeds.2017.11.057

**Published:** 2018-04

**Authors:** Miia Lehtovirta, Katja Pahkala, Harri Niinikoski, Antti J. Kangas, Pasi Soininen, Hanna Lagström, Jorma S.A. Viikari, Tapani Rönnemaa, Antti Jula, Mika Ala-Korpela, Peter Würtz, Olli T. Raitakari

**Affiliations:** 1 Research Centre of Applied and Preventive Cardiovascular Medicine, University of Turku, Turku, Finland; 2 Department of Pediatrics and Adolescent Medicine, Turku University Hospital, Turku, Finland; 3 Computational Medicine, Faculty of Medicine, University of Oulu & Biocenter Oulu, Oulu, Finland; 4 Department of Public Health, University of Turku, Turku, Finland; 5 Department of Medicine, University of Turku and Division of Medicine, Turku University Hospital, Turku, Finland; 6 National Institute for Health and Welfare, Turku, Finland; 7 Medical Research Council Integrative Epidemiology Unit at the University of Bristol, Bristol, United Kingdom; 8 NMR Metabolomics Laboratory, School of Pharmacy, University of Eastern Finland, Kuopio, Finland; 9 Population Health Science, Bristol Medical School, University of Bristol, Bristol, United Kingdom; 10 Systems Epidemiology, Baker Heart and Diabetes Institute; 11 Department of Epidemiology and Preventive Medicine, School of Public Health and Preventive Medicine, Faculty of Medicine, Nursing and Health Sciences, The Alfred Hospital, Monash University, Melbourne, Victoria, Australia; 12 Department of Clinical Physiology and Nuclear Medicine, Turku University Hospital, Turku, Finland

**Keywords:** diet, fatty acids, metabolomics, metabolic profiling, primordial prevention, BMI, Body mass index, E%, Percentage of total energy intake, HDL, High-density lipoprotein, HDL-C, High-density lipoprotein cholesterol, IDL, Intermediate-density lipoprotein, LDL, Low-density lipoprotein, LDL-C, Low-density lipoprotein cholesterol, MUFA, Monounsaturated fatty acids, NMR, Nuclear magnetic resonance, PUFA, Polyunsaturated fatty acids, SAFA, Saturated fatty acids, STRIP, The Special Turku Coronary Risk Factor Intervention Project, VLDL, Very low-density lipoprotein

## Abstract

**Objectives:**

To study the effects of repeated, infancy-onset dietary counseling on a detailed metabolic profile. Effects of dietary saturated fat replacement on circulating concentrations of metabolic biomarkers still remain unknown.

**Study design:**

The Special Turku Coronary Risk Factor Intervention Project (STRIP) study is a longitudinal, randomized atherosclerosis prevention trial in which repeated dietary counseling aimed at reducing the proportion of saturated fat intake. Nuclear magnetic resonance metabolomics quantified circulating metabolites from serum samples assessed at age 9 (n = 554), 11 (n = 553), 13 (n = 508), 15 (n = 517), 17 (n = 457), and 19 (n = 417) years.

**Results:**

The intervention reduced dietary intake of saturated fat (mean difference in daily percentage of total energy intake: −2.1 [95% CI −1.9 to −2.3]) and increased intake of polyunsaturated fat (0.6 [0.5-0.7]). The dietary counseling intervention led to greater serum proportions of polyunsaturated fatty acids (*P* < .001), with greater proportions of both circulating omega-3 (*P* = .02) and omega-6 (*P* < .001) fatty acids. The proportion of saturated fatty acids in serum was lower for both boys and girls in the intervention group (*P* < .001), whereas the serum proportion of monounsaturated fat was lower for boys in the intervention group only (*P* < .001). The intervention also reduced circulating intermediate-density lipoprotein and low-density lipoprotein lipid concentrations (*P* < .01). Dietary intervention effects on nonlipid biomarkers were minor except from greater concentrations of glutamine in the intervention group.

**Conclusions:**

Repeated dietary counseling from infancy to early adulthood yielded favorable effects on multiple circulating fatty acids and lipoprotein subclass lipids, particularly in boys. These molecular effects substantiate the beneficial role of saturated fat replacement on the metabolic risk profile.

**Trial registration:**

ClinicalTrials.gov: NCT00223600.

The development of atherosclerosis is a lifelong process that starts in childhood and leads to cardiovascular complications in later life.^1^, ^2^, ^3^ Efforts to prevent atherosclerosis at an early age are therefore justified to inhibit the establishment of cardiovascular risk factors. Central to this primordial prevention is a prudent diet aimed to prevent the development of adverse lipid levels, excess adiposity, and elevated blood pressure.^4^, ^5^

The Special Turku Coronary Risk Factor Intervention Project (STRIP, ClinicalTrials.gov: NCT00223600) was conducted to study the effect of dietary intervention initiated in infancy and maintained until the age of 20 years on cardiometabolic risk factors.^6^ It is the only randomized trial examining the health effects of reduced saturated fat diet in healthy individuals from infancy to young adulthood.^1^, ^6^ The intervention comprised repeated, individualized dietary counseling with a main focus on replacing intake of saturated fat with unsaturated fat. We have shown previously that the intervention is effective in decreasing saturated fat intake and leads to lower serum low-density lipoprotein cholesterol (LDL-C) concentration from infancy until 19 years of age.^7^ In addition, the intervention has been associated with improved insulin sensitivity,^8^ lower blood pressure,^9^ enhanced brachial artery endothelial function,^2^ increased ideal cardiovascular health score,^10^ and reduced risk for the metabolic syndrome.^11^ What is currently not well understood is the influence of reduced saturated fat intake on circulating levels of fatty acids and lipoprotein subclass measures. The dietary counseling also potentially could affect even nonlipid metabolites, such as amino acid levels and other emerging metabolic biomarkers for the risk of cardiometabolic diseases.^12^

The present study was undertaken to assess the effects of the dietary-counseling intervention on a detailed serum metabolic profile. High-throughput nuclear magnetic resonance (NMR) metabolomics provides simultaneous quantification of circulating metabolites, including fatty acids, amino acids, and detailed lipoprotein subclass profiling. This methodology has uncovered and validated novel fatty acid and nonlipid biomarkers for the risk of cardiovascular disease and type 2 diabetes.^12^, ^13^, ^14^, ^15^, ^16^ The detailed metabolic profiling can further provide increased molecular understanding of the pathophysiology of atherosclerosis and underlying risk factors.^17^, ^18^, ^19^, ^20^ Here, we investigated the effects of the randomized STRIP dietary intervention trial on a comprehensive serum metabolic profile measured at 6 time points at ages 9, 11, 13, 15, 17, and 19 years in 554 participants.

## Methods

**Figure 1:**
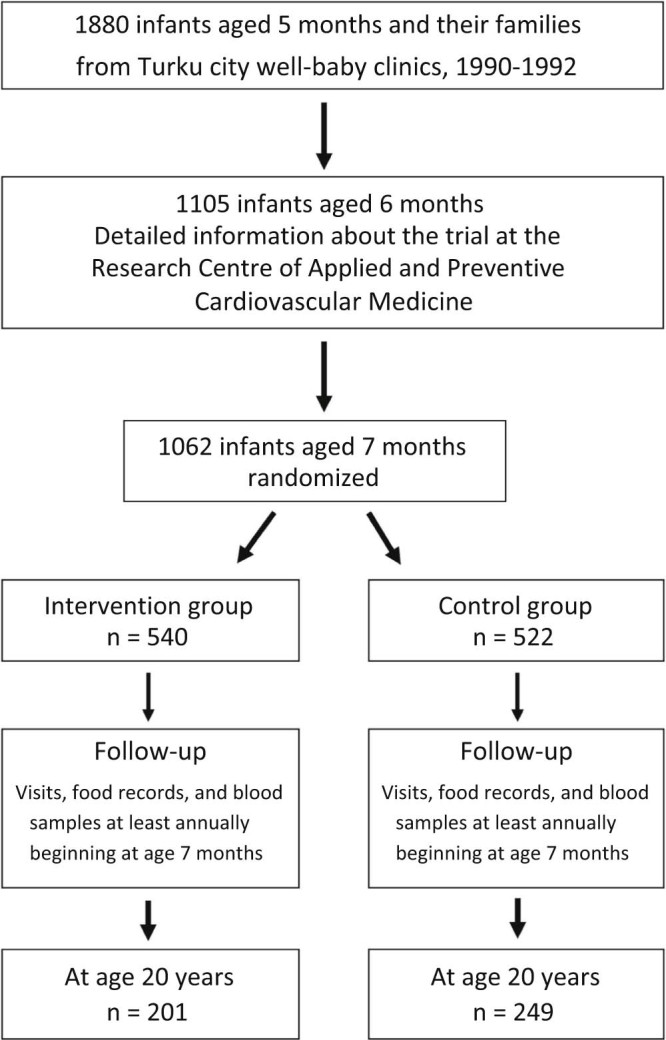
Flowchart of the STRIP study.

The STRIP study is a prospective, randomized, infancy-onset intervention trial aiming to reduce the risk of atherosclerosis.^6^, ^7^, ^11^ The families of 5-month-old infants were recruited to the study from well-baby clinics in Turku, Finland, between February 1990 and June 1992. When the infants were 6 months old, their families received detailed information about STRIP, and a total of 1062 infants (56.5% of the eligible age cohort) then embarked on the study (Figure 1; available at www.jpeds.com). At the age of 7 months, they were allocated randomly to a dietary intervention group (N = 540; 256 girls) or a control group (N = 522; 256 girls). Both groups met with a nutritionist and a pediatrician or a nurse during their study visits.

The intervention group received individualized dietary counseling at 1- to 3-month intervals until the child was 2 years of age and biannually thereafter until 20 years of age.^6^, ^11^, ^21^ The children in the control group received only basic health education routinely given at Finnish well-baby clinics and by school health care. The control group was met biannually until 7 years of age and annually thereafter until 20 years of age.

For the present analysis, data were available from 6 time points with metabolic biomarkers quantified by high-throughput NMR metabolomics from serum samples drawn at age 9 (n = 554), 11 (n = 553), 13 (n = 508), 15 (n = 517), 17 (n = 457), and 19 (n = 417) years. This represents 92%-99% of total number of study participants. This kind of an intense intervention trial spanning over 2 decades inevitably has a substantial loss to follow-up. No systematic differences have been found between the study participants and those lost to follow-up in key characteristics, such as weight, total cholesterol, blood pressure, or saturated fat intake.^2^, ^6^, ^11^ The study was approved by the Joint Commission on Ethics of the Turku University and the Turku University Central Hospital. Written informed consent was obtained from the parents in the beginning of the study and from the adolescents at 15 and 18 years of age.

### Dietary Counseling and Food Records

The individualized dietary counseling was designed to meet the Nordic Dietary Recommendations.^22^, ^23^ The main aim was to replace saturated fat with unsaturated fat in the diet without reducing the total fat intake. The intervention aimed at fat intake of 30%-35% of daily energy (E%, or percentage of total energy intake), unsaturated to saturated fatty acid (SAFA) ratio of 2:1, and cholesterol intake of <200 mg/d. A fixed diet was never ordered but instead changes in the diet were suggested based on the child's food records (eg, replacement of dairy fat–blend spreads with vegetable oil–based spreads).

In the beginning of the intervention trial, breast feeding or formula was advised until 1 year of age, and after that, 0.5-0.6 L of skimmed milk daily was recommended for the intervention children. The intervention families were advised to add 2-3 teaspoonfuls of soft margarine or vegetable oil to the child's diet daily from 12 to 24 months of age. Quality of dietary fat was a major topic of the counseling throughout childhood and adolescence but the use of vegetables, fruits, and low-salt and whole-grain products also was recommended.^11^, ^24^ In terms of protein, specific counseling related to plant- or animal-based sources was not given. The counseling was given to the parents until the child was 7 years of age, and from then onward, gradually more information was given directly to the child. Most of the counseling material used, eg, brochures and paper-pencil tasks, was especially developed for the project due to the lack of ready-made materials for children. The parents were informed about the contents of the child's counseling sessions and encouraged to discuss the topics with the child at home.

All families (parents, caregivers) and school staff kept food records of the child's food intake. A 3-day food record was obtained every 6 months until the age of 2 years and after that, a 4-day food record was used to account for greater variation in the diet. After 7 years, the intervention children continued to keep food records biannually, and the control children kept them annually. Food records were kept for consecutive days, and they included at least 1 weekend day. During follow-up visits, the nutritionist reviewed the food records for completeness and accuracy. Nutrient intakes were analyzed with a Micro Nutrica program, developed by the Research and Development Centre of the Social Insurance Institution, Turku, Finland.^25^ The data bank of the program is flexible, permitting continuous updating and additions of new single or composite foods.

### Lipid and Metabolite Quantification

A high-throughput NMR metabolomics platform was used for quantification of 60 serum lipid and metabolite measures (Table I; available at www.jpeds.com). This platform provides measurement of routine lipids, lipid concentrations of 14 lipoprotein subclasses and major subfractions, and further quantifies abundant fatty acids, amino acids, ketone bodies, and gluconeogenesis-related metabolites in absolute concentration units.^13^ The metabolic profiling was measured from fasting serum samples from 6 time points collected when the study participants were aged 9-19 years (Table II; available at www.jpeds.com). The NMR metabolomics platform recently has been used in various epidemiologic studies^20^, ^26^, ^27^ and details of the experimentation have been described.^13^, ^28^

### Statistical Analyses

Metabolic measures with skewed distributions were log(x+1)-transformed before analyses. Analyses were conducted both sex-combined and stratified by sex because the STRIP study has previously reported pronounced sex interactions in the lipid-lowering effects of the intervention.^7^ For each measure of metabolite concentrations or dietary intake, a linear mixed-effects model for repeated measures was fitted with the intervention group, sex and age as fixed effects and subject as a random effect. Analysis with study group×sex interaction included in the model also were performed. To facilitate comparison of effect sizes across metabolites, all metabolic measures were scaled to SD units. The effect sizes of the intervention reported hereby correspond to the average difference in SD-scaled metabolite concentration due to the intervention. Age×study group interaction was included in all dietary intake analyses. Statistical analyses were conducted with R 3.2 software (R Foundation for Statistical Computing, Vienna, Austria).^29^

## Results

This study investigated the effect of a randomized, infancy-onset dietary intervention on the circulating metabolic profile of 417-554 participants assessed at 6 time points between 9 and 19 years of age. Clinical characteristics of the study participants are shown in Table II.

### Intervention Effects on Dietary Fat Intake

**Figure 2:**
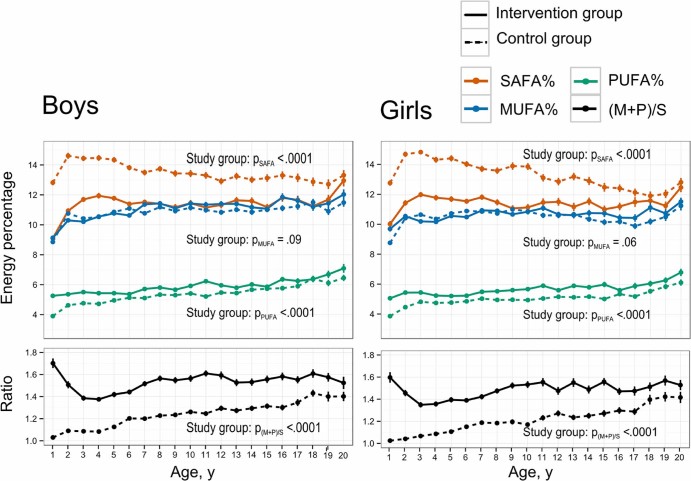
Dietary intake of fatty acids in the intervention and control groups of boys and girls. Values are mean (SE) dietary intake in energy percentage of saturated, monounsaturated, and polyunsaturated fatty acids from age 1 to 20 years. The dietary intake is also shown for the sum of MUFA + PUFA relative to SAFA.

Dietary intake of different fatty acid types for boys and girls in the intervention and control groups is shown in Figure 2. Children in the intervention group had lower saturated fat intake compared with control children throughout the intervention. However, the difference of approximately 3 E% in early childhood decreased toward the end of the intervention at 20 years of age (mean difference in daily E% between groups: −2.0 [95% CI −1.7 to −2.3] in boys and −1.8 [−1.5 to −2.1] in girls). The intervention also resulted in greater intakes of polyunsaturated fatty acids (PUFA) for both sexes (mean daily difference in E% 0.5 [0.4-0.6] for boys and 0.6 [0.5-0.8] for girls), but this difference decreased toward adolescence. Consequently, the intervention children had a greater ratio of unsaturated to saturated fat in their diet compared with control children throughout the intervention. For both boys and girls, no consistent differences were observed in the dietary intake of monounsaturated fatty acids (MUFAs).

### Intervention Effects on Serum Metabolic Profile

**Figure 3:**
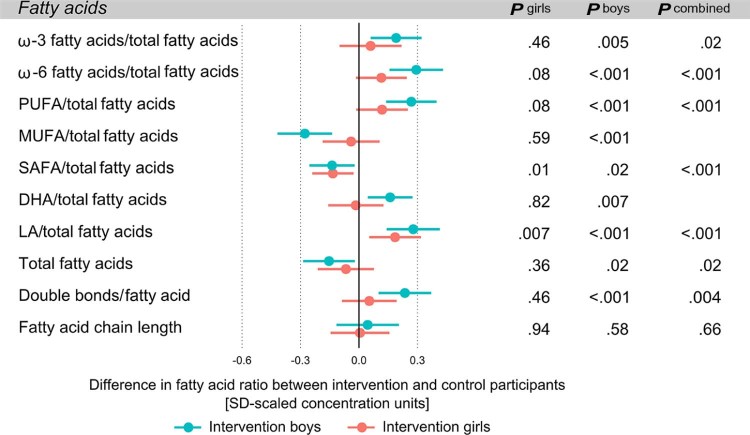
Differences in circulating fatty acid ratios between the intervention group and the control group. Effect estimates are SD-scaled differences between boys in the intervention group with respect to boys in the control group (*blue*) and girls in the intervention group with respect to girls in the control group (*red*). *Error bars* indicate 95% CIs. *P* values for sex-combined analysis are shown in case there were no statistically significant study group×sex interaction. Metabolic measures are from pooled analyses across the 6 time points. *DHA*, docosahexaenoic acid; *LA*, linoleic acid; *ω-3 fatty acids/total fatty acids*, ratio of omega-3 fatty acids to total fatty acids.

**Figure 4:**
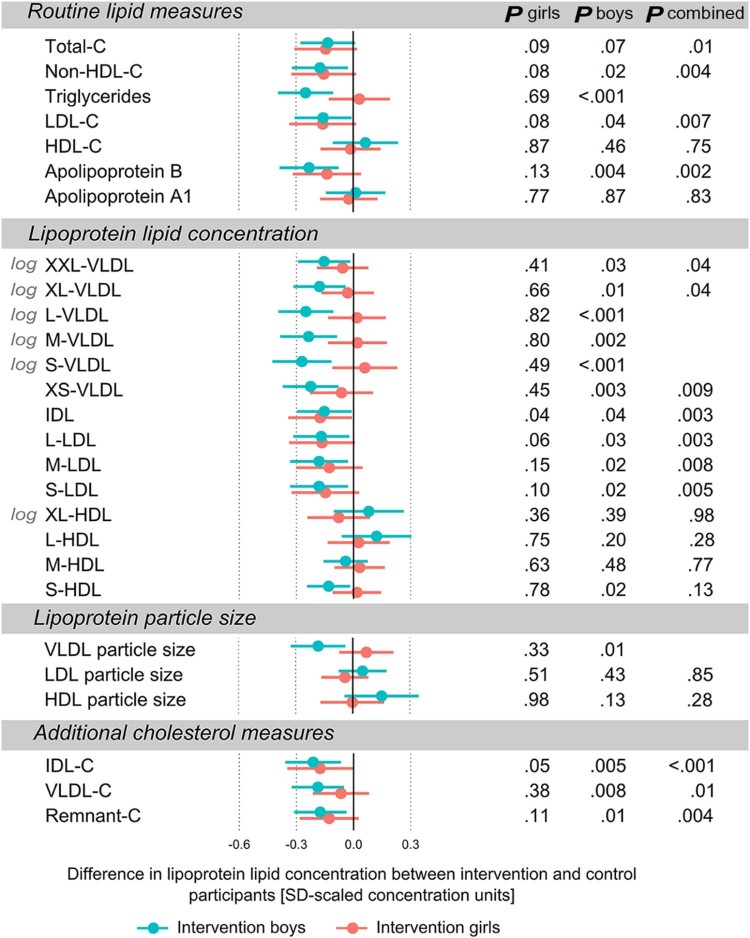
Differences in serum lipid measures between the intervention group and the control group. Effect estimates are SD-scaled differences between boys in the intervention group with respect to boys in the control group (*blue*) and girls in the intervention group with respect to girls in the control group (*red*). *Error bars* indicate 95% CIs. *P* values for sex-combined analysis are shown in case there were no statistically significant study group×sex interaction. Lipid measures are from pooled analysis across the 6 time points, and those with skewed distributions were log(x+1)-transformed before analyses. *C*, cholesterol; *L*, large, *M*, medium; *S*, small; *XL*, very large; *XS*, very small; *XXL*, extremely large.

**Figure 5:**
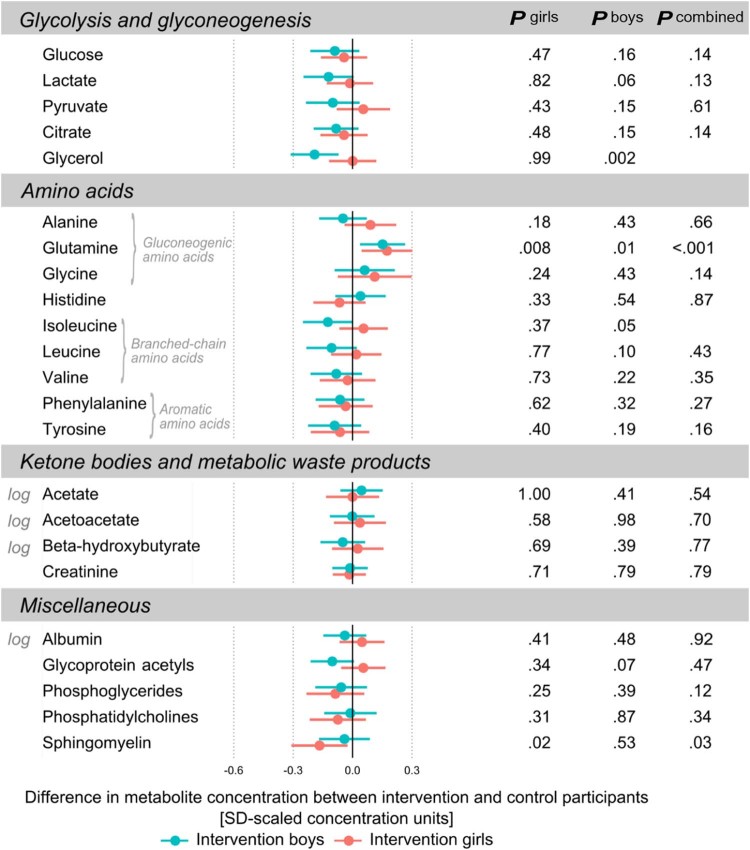
Differences in circulating metabolites between the intervention group and the control group. Effect estimates are SD-scaled differences between boys in the intervention group with respect to boys in the control group (*blue*) and girls in the intervention group with respect to girls in the control group (*red*). *Error bars* indicate 95% CIs. *P* values for sex-combined analysis are shown in case there were no statistically significant study group×sex interaction. Metabolic measures are from pooled analyses across the 6 time points, and those with skewed distributions were log(x+1)-transformed before analyses.

The effects of the dietary intervention on circulating metabolic profile are illustrated separately for boys and girls in Figure 3, Figure 4, Figure 5. Overall, the metabolic effects of the intervention tended to be stronger in boys than in girls but generally following a similar pattern in both sexes. All differences between the intervention group and controls are shown in SD-scaled concentration units to enable comparison across the metabolic measures. The corresponding differences in absolute concentrations are listed in Table I.

#### Serum Fatty Acids

The most prominent differences between the intervention group and the control group were observed in circulating fatty acids (Figure 3). In boys, the serum proportion of PUFA, relative to total fatty acids, was greater in the intervention group compared with the control group (0.30 SD [0.14-0.40], corresponding to 1.0% [0.53-1.49] greater PUFA ratio). Greater serum proportions were observed for both PUFA omega-3 and omega-6 fatty acids, but the effects tended to be stronger for omega-6. Similar effects were observed for the main constituents of these PUFAs, namely docosahexaenoic acid (an omega-3 fatty acid) and linoleic acid (an omega-6 fatty acid). In contrast, the serum MUFA proportion was lower among the intervention group compared with controls. In girls, the difference in PUFA proportion between the intervention and control groups were weaker than in boys, but the association pattern was similar, which is reflected as a strong combined analysis result. There was a significant sex interaction for the proportion of MUFA and docosahexaenoic acid. The proportion of serum SAFAs was significantly lower in the intervention group compared with the control group.

#### Lipoprotein Lipids

The dietary intervention effects on a detailed panel of lipoprotein lipid measures are shown in Figure 4. The intervention effects on the routine lipid measures, eg, LDL-C, are shown to facilitate interpretation and put the association magnitudes into perspective. We have shown that the repeated dietary counseling given in the STRIP study is associated with decreased LDL-C concentrations in both boys and girls.^7^ In the present study, the levels of total cholesterol, non–high-density lipoprotein cholesterol (HDL-C), and LDL-C were reduced for both sexes, whereas the concentrations of triglycerides and apolipoprotein B were reduced for the intervention boys only. No differences were found between the concentrations of HDL-C and apolipoprotein A-I between the study groups. The current analyses of lipoprotein subclasses were undertaken to provide more insight into the detailed lipid effects of the intervention. Overall, the lipoprotein subclass profiling showed reduced lipid concentrations (ie, combined concentration of cholesterol, phospholipid, and triglycerides) in the very low-density lipoprotein (VLDL) particles in the intervention boys and reduced lipid concentrations in the intermediate-density lipoprotein (IDL) and LDL subclasses in the intervention group. In the intervention boys, the lipid concentrations in VLDL particles were significantly lower, in particular for medium-sized VLDL (−0.24 SD [−0.39 to −0.09]) and small VLDL (−0.27 SD [−0.42 to −0.12]). Accordingly, the levels of remnant-cholesterol (=VLDL cholesterol + IDL cholesterol) were lower for boys in the intervention group. Also, VLDL particle size was smaller for boys in the intervention group. No effects for VLDL lipids were observed for girls. There were no differences found in serum triglycerides between the girls in the intervention group and the girls in the control group, and this might be reflected in VLDL particles because triglycerides are their main constituents. For both sexes, no robust differences were observed in the lipid concentration of HDL subclasses, although a tendency of lower lipid concentration of small HDL particles was evident in the intervention boys compared with controls.

#### Nonlipid Metabolites

The intervention effects on nonlipid metabolic biomarkers were modest and broadly similar for boys and girls (Figure 5). The most robust metabolic effect was found for greater levels of glutamine in the intervention group compared with the control children. Furthermore, boys in the intervention group also had lower serum levels of glycerol and a tendency toward lower levels of branched-chain amino acids and glycoprotein acetyls, an inflammatory biomarker,^30^ than the boys in the control group.

## Discussion

Repeated dietary counseling on low saturated fat intake from infancy to early adulthood yielded favorable metabolic effects on multiple circulating fatty acids that are predictive of cardiovascular event risk and type 2 diabetes. The greater dietary intake of PUFA in the intervention group resulted in greater blood proportion of both serum omega-3 and omega-6 PUFAs. The intervention group had lower dietary intake of SAFA, and the circulating SAFA proportion also was reduced in the intervention group. In contrast to dietary intake, the MUFA proportion was decreased in intervention boys. All these differences in the serum fatty acid balance are consistent with a less risk-prone profile for cardiovascular diseases and type 2 diabetes.^13^, ^14^, ^16^ These results extend previous findings from the STRIP study demonstrating lowering of serum LDL-C in the intervention group^7^—a conclusion further corroborated here by lipoprotein subclass profiling and extended also to VLDL and IDL cholesterol. The intervention had limited effects on nonlipid metabolites, with increased levels of glutamine as the only robust exception. The overall molecular effects assessed at 6 time points in a long-running dietary intervention trial substantiate the beneficial role of replacing SAFA intake on the detailed metabolic risk profile.

Dietary guidelines recommend the reduction of SAFA intake to lower the risk of cardiovascular diseases.^31^, ^32^ Unsaturated fats are the preferred replacement nutrient, especially PUFAs. Accordingly, the intervention reduced the dietary intake of SAFA and increased the intake of PUFA in both sexes. Many observational studies have linked greater circulating levels of omega-3 fatty acids with reduced cardiovascular disease risk.^14^, ^33^ However, clinical trial evidence is unsupportive on benefits of omega-3 fatty acid supplementation.^34^, ^35^ Novel reports in large prospective cohorts^14^, ^36^ have strengthened the evidence that greater levels of circulating omega-6 PUFAs predict reduced cardiovascular risk; however, causality remains unclear. Serum levels of MUFA are rarely measured in large studies, but a recent meta-analysis of 3 prospective studies demonstrated adverse cardiovascular risk association for greater MUFA, and the MUFA to be as strong biomarker as LDL-C.^14^ Whereas this might be considered to contrast dietary recommendations on the benefit in MUFA intake, the present study demonstrates that dietary MUFA intake and circulating serum levels of MUFA do not follow a similar pattern. Although we cannot separate whether the decrease in MUFA ratio observed for intervention boys is a result of increased PUFA intake or decreased SAFA intake, we speculate that lower serum proportion of MUFA is primarily due to decreased SAFA intake, because desaturation from SAFA is a primary source for serum MUFAs.^26^ It is also reported that dietary SAFA and MUFA intakes correlate in Western countries.^37^, ^38^

The STRIP study previously has shown that the dietary intervention leads to reduction in serum total and LDL-C, as well as triglycerides and apolipoprotein B in boys from infancy until 19 years of age.^7^ The difference in LDL-C also was seen in girls when analyzed between the ages of 5 and 19 years.^7^, ^39^ In the present analysis, we examined the differences in the metabolic profile between the study groups in 6 time points. The benefits of the intervention are substantiated by our results on the more detailed lipoprotein subclass patterns, with reduced lipid concentrations in LDL and IDL particles for both sexes and in the boys in the intervention group also in the small and medium-sized VLDL. These VLDL lipoprotein particles are the main carriers of cholesterol in the VLDL fraction and may be small enough to penetrate the arterial wall to cause atherosclerosis.^40^ Consistent with these results, the cholesterol carried by VLDL and IDL particles, ie, remnant cholesterol, was lower in the boys in the intervention group compared with the control boys. Evidence in humans shows that remnant cholesterol may have a causal role in development of atherosclerosis and the risk for ischemic heart disease.^40^ In line with our results, an intervention study targeting the intake of whole grains, fish, and bilberries in adults with impaired glucose metabolism found changes in lipid metabolites, eg, increased concentration of large high-density lipoprotein particle (L-HDL) particles.^19^

Many nonlipid metabolites, such as amino acids, are emerging biomarkers for the risk of cardiovascular disease and type 2 diabetes; however, the underlying mechanisms are still largely unclear.^12^, ^14^, ^26^ For instance, greater serum glutamine levels are predictive of lower risk for type 2 diabetes.^41^ Glutamine also has been associated inversely with cardiovascular disease risk.^14^ Branched-chain amino acids, including isoleucine, are associated with insulin resistance and risk for type 2 diabetes.^16^, ^41^ Although the intervention effect on these nonlipid metabolites was mostly weak, the metabolic differences were in the direction of lower cardiometabolic risk, especially for boys in the intervention group. We have shown previously that serum insulin is lower in both boys and girls in the intervention group. However, there were only minor effects on the gluconeogenesis-related metabolites, albeit a tendency for lower concentrations for boys in the intervention group were observed.

To make the reported metabolic differences more concrete, it is worthwhile to compare the results to recently reported causal effects of body mass index (BMI) on metabolic profile in young adults.^18^ For instance, the dietary intervention resulted in 0.30 SD greater serum proportion of omega-6 fatty acid, ie, ratio of omega-6 fatty acids concentration to total fatty acid concentration, for boys and 0.15 SD greater for girls—this effect is similar to the causal effect of ≈7 lower BMI units (kg/m^2^) for boys and ≈3 lower BMI units for girls. Similarly, the effect of the intervention on LDL-C corresponds to the causal effect of ≈2 units lower BMI for both sexes. Although we acknowledge that adiposity has wider metabolic effects than the dietary effects observed here,^18^ these comparisons nevertheless give perspective to the notable magnitude of the metabolic effects of the intervention.

Overall, the effect of the dietary intervention on the metabolic profile was more prominent in boys than in girls, even though the differences in dietary intakes of fatty acids were similar in both sexes. The sex difference is most evident in triglycerides and reflected in the lipid concentrations of VLDL particles. This discrepancy in serum lipids has been reported in our previous studies and the reasons are still unknown.^7^, ^39^, ^42^ Differences in body composition, a more pronounced intervention effect on consumption of favored/unfavored foods in boys, and sex hormone levels may have an impact on this outcome.

The strengths and limitations of the study should be considered. The STRIP trial is unique worldwide, as no other study has introduced dietary counseling in infancy and continued it for 20 years. However, the long-term intervention study design limits options for comparison to similar studies and replication. Nevertheless, the observed effects of dietary intake and corresponding circulating fatty acid differences are supported by observational cohorts.^14^ Strengths of the study include detailed metabolic profiling of a large number of participants repeated at up to 6 time points studied, which allowed us to uncover molecular effects of the intervention across many established and emerging metabolic biomarkers. Limitations include potential selection bias during the initial recruitment phase as the participating families might have been more interested in health-related matters. The intervention effect might also have been diluted because the control children and their families regularly received information regarding, for example, their serum cholesterol levels. This kind of an intervention trial spanning over 2 decades inevitably has a substantial loss to follow-up. The most common reasons for discontinuance were moving away from the area, at young age child's recurring infections, and reluctance to blood sampling.^6^ No systematic differences have been found between the study participants and those lost to follow-up in key characteristics, such as weight, total cholesterol, blood pressure, or saturated fat intake.^2^, ^6^, ^11^ Therefore, our results were unlikely influenced by systematic selection bias. A limitation of the study is also that we do not have hormonal or body composition data to allow detailed analyses into the sex differences. The dietary intervention in the STRIP study had prominent metabolic effects on serum of fatty acid balance, in concordance with dietary SAFA replacement, which led to greater intake of PUFA. The proportions of serum omega-3 and omega-6 fatty acids were greater in the intervention group compared with the control group. The proportion of circulating MUFA was lower in the intervention group, partly contrasting the dietary intake. However, the circulating fatty acid differences were in line with lower cardiovascular disease risk and type 2 diabetes risk. This was especially seen in boys, whereas metabolic effects in girls were less prominent. The levels of total cholesterol, non–HDL-C, and LDL-C were reduced in the intervention group for both sexes. Boys also displayed favorable lowering of triglycerides and triglyceride-rich lipoprotein subclasses in the intervention group. These results emphasize the value of detailed metabolic profiling to assess the molecular effects of dietary supplementation or counseling.^43^ The circulating metabolic biomarkers might serve as better markers of long-term dietary quality and could present as molecular intermediates to bridge the intricate relation between dietary fat and cardiovascular risk. To conclude, the STRIP dietary intervention resulting in lower saturated fat intake was favorably reflected in multiple serum fatty acids and lipoprotein subclasses through childhood to young adulthood. Future follow-ups will show whether these changes lead to lower cardiovascular event risk.

## Figures and Tables

**Table I:** Mean (SD) metabolite concentrations and effect estimates for all metabolic measures analyzed in absolute concentration units

Metabolite measures	Girls Mean (SD)	Boys Mean (SD)	Girls effect estimate, beta	Boys effect estimate, beta
Omega-3 fatty acids, %	4.21 (0.91)	4.12 (0.84)	0.05240	0.16700
Omega-6 fatty acids, %	34.05 (3.22)	34.09 (3.36)	0.37900	0.96900
PUFA, %	38.44 (3.68)	38.29 (3.80)	0.44400	1.01000
MUFA, %	23.84 (2.69)	23.62 (2.91)	−0.11300	−0.78100
SAFA, %	37.90 (2.45)	38.17 (2.57)	−0.33700	−0.34700
Docosahexaenoic acid, %	1.72 (0.65)	1.55 (0.55)	−0.00971	0.09660
Linoleic acid, %	27.44 (3.27)	27.34 (3.41)	0.61900	0.93100
Total fatty acids, mmol/L	9.19 (1.99)	8.56 (1.97)	−0.13400	−0.30800
Degree of unsaturation	1.19 (0.07)	1.18 (0.06)	0.00348	0.01550
Total cholesterol, mmol/L	3.93 (0.87)	3.64 (0.82)	−0.12300	−0.11400
Non–HDL-C, mmol/L	2.53 (0.79)	2.32 (0.75)	−0.11900	−0.13500
Total triglycerides, mmol/L	0.84 (0.35)	0.77 (0.34)	0.01110	−0.08600
LDL-C, mmol/L	1.37 (0.45)	1.24 (0.42)	−0.07060	−0.06950
HDL-C, mmol/L	1.41 (0.27)	1.32 (0.29)	−0.00378	0.01810
Apolipoprotein B, g/L	0.74 (0.15)	0.70 (0.15)	−0.02100	−0.03530
Apolipoprotein A-1, g/L	1.44 (0.18)	1.37 (0.17)	−0.00414	0.00237
log extremely large VLDL, mmol/L	0.02 (0.02)	0.02 (0.02)	−0.00103	−0.00279
log very large VLDL, mmol/L	0.03 (0.04)	0.03 (0.04)	−0.00128	−0.00740
log large VLDL, mmol/L	0.12 (0.10)	0.12 (0.11)	0.00189	−0.02670
log medium VLDL, mmol/L	0.30 (0.12)	0.29 (0.13)	0.00260	−0.03030
log small VLDL, mmol/L	0.33 (0.10)	0.31 (0.09)	0.00562	−0.02560
Very small VLDL, mmol/L	0.41 (0.11)	0.38 (0.11)	−0.00689	−0.02450
IDL, mmol/L	0.97 (0.27)	0.89 (0.25)	−0.04590	−0.04020
Large LDL, mmol/L	1.08 (0.31)	0.98 (0.29)	−0.05050	−0.05170
Medium LDL, mmol/L	0.59 (0.18)	0.54 (0.17)	−0.02260	−0.03210
Small LDL, mmol/L	0.38 (0.11)	0.35 (0.10)	−0.01610	−0.01980
log very large HDL, mmol/L	0.41 (0.12)	0.37 (0.14)	−0.00996	0.01030
Large HDL, mmol/L	0.78 (0.26)	0.69 (0.28)	0.00746	0.03350
Medium HDL, mmol/L	0.78 (0.17)	0.74 (0.14)	0.00512	−0.00661
Small HDL, mmol/L	0.98 (0.13)	0.96 (0.11)	0.00210	−0.01550
VLDL particle size, nm	35.99 (1.17)	36.05 (1.30)	0.08900	−0.22600
LDL particle size, nm	23.69 (0.19)	23.69 (0.22)	−0.00835	0.01040
HDL particle size, nm	10.08 (0.21)	10.02 (0.24)	−5.12E−04	0.03430
IDL cholesterol, mmol/L	0.62 (0.18)	0.57 (0.16)	−0.02982	−0.03601
VLDL cholesterol, mmol/L	0.54 (0.20)	0.50 (0.21)	−0.01391	−0.03919
Remnant cholesterol, mmol/L	1.61 (0.37)	1.08 (0.37)	−0.04792	−0.06558
Glucose, mmol/L	3.90 (0.38)	4.00 (0.42)	−0.01730	−0.03610
Lactate, mmol/L	1.34 (0.37)	1.39 (0.39)	−0.00507	−0.04630
Pyruvate, mmol/L	0.06 (0.02)	0.06 (0.02)	0.00117	−0.00214
Citrate, mmol/L	0.10 (0.02)	0.10 (0.02)	−8.24E−04	−0.00159
Glycerol, mmol/L	0.07 (0.03)	0.07 (0.02)	1.68E−05	−0.00485
Alanine, mmol/L	0.34 (0.06)	0.33 (0.06)	0.00524	−0.00281
Glutamine, mmol/L	0.50 (0.07)	0.51 (0.08)	0.01320	0.01150
Glycine, mmol/L	0.25 (0.05)	0.25 (0.04)	0.00480	0.00263
Histidine, mmol/L	0.06 (0.01)	0.06 (0.01)	−6.09E−04	3.70E−04
Isoleucine, mmol/L	0.05 (0.01)	0.05 (0.01)	6.71E−04	−0.00150
Leucine, mmol/L	0.07 (0.01)	0.08 (0.01)	2.44E−04	−0.00138
Valine, mmol/L	0.17 (0.03)	0.18 (0.03)	−8.07E−04	−0.00263
Phenylalanine, mmol/L	0.07 (0.01)	0.06 (0.01)	−3.05E−04	−5.54E−04
Tyrosine, mmol/L	0.05 (0.01)	0.05 (0.01)	−6.60E−04	−9.46E−04
log acetate, mmol/L	0.04 (0.01)	0.04 (0.01)	−8.58E−07	5.34E−04
log acetoacetate, mmol/L	0.05 (0.05)	0.06 (0.05)	0.00194	−8.31E−05
log 3-hydroxybutyrate, mmol/L	0.11 (0.08)	0.11 (0.07)	0.00193	−0.00363
Creatinine, mmol/L	0.04 (0.01)	0.05 (0.01)	−1.98E−04	−1.54E−04
log albumin, cu	0.09 (0.01)	0.09 (0.01)	3.32E−04	−2.72E−04
Glycoprotein acetyls, mmol/L	1.22 (0.24)	1.19 (0.22)	0.01250	−0.02350
Total phosphoglycerides, mmol/L	1.69 (0.40)	1.54 (0.35)	−0.03290	−0.02180
Phosphatidylcholines, mmol/L	1.63 (0.36)	1.50 (0.33)	−0.02600	−0.00382
Sphingomyelins, mmol/L	0.41 (0.09)	0.37 (0.09)	−0.01580	−0.00387

Mean and SD concentrations of the metabolic measures are from pooled analyses across the 6 time points are shown. A total of 60 metabolic measures were quantified via a high-throughput serum NMR metabolomics platform.^14^ Quantification of lipoprotein subclasses was calibrated against high-performance liquid chromatography on an external set of samples. The 14 lipoprotein subclass sizes were defined as follows: extremely large VLDL with particle diameters from 75 nm upwards and a possible contribution of chylomicrons, 5 VLDL subclasses (average particle diameters of 64.0 nm, 53.6 nm, 44.5 nm, 36.8 nm, and 31.3 nm), IDL (28.6 nm), 3 LDL subclasses (25.5 nm, 23.0 nm, and 18.7 nm), and 4 HDL subclasses (14.3 nm, 12.1 nm, 10.9 nm, and 8.7 nm). The mean size for VLDL, LDL, and HDL particles was calculated by weighting the corresponding subclass diameters with their particle concentrations.

**Table II:** Characteristics of the study participants at 9 and 19 years of age

Characteristics	9 y				19 y			
	Girls (n = 268)		Boys (n = 286)		Girls (n = 211)		Boys (n = 206)	
	I	Ctl	I	Ctl	I	Ctl	I	Ctl
Height, cm	135.6 (5.9)	135.5 (5.8)	136.0 (5.9)	136.5 (5.3)	167.7 (6.2)	167.1 (6.1)	181.1 (6.6)	181.5 (6.1)
Weight, kg	31.1 (5.5)	31.7 (6.9)	30.6 (5.1)	31.1 (5.2)	62.6 (10.6)	63.4 (12.8)	72.8 (11.4)	75.2 (12.0)
BMI, kg/m^2^	16.8 (2.1)	17.1 (2.8)	16.5 (1.9)	16.6 (2.2)	22.2 (3.3)	22.7 (4.4)	22.2 (3.0)	22.9 (3.7)
Overweight, %	15.6	22.1	13.1	13.4	9.7	18.6	16.3	22.2
Systolic blood pressure, mm Hg	100.8 (7.7)	100.7 (8.4)	99.8 (6.5)	100.5 (6.5)	116.2 (10.2)	115.1 (12.6)	126.9 (12.1)	129.0 (13.4)
Diastolic blood pressure, mm Hg	57.6 (5.7)	58.2 (6.2)	57.3 (5.7)	58.1 (5.9)	64.9 (6.3)	65.4 (7.7)	65.9 (7.8)	66.3 (8.2)
Dietary energy intake, kcal	1555 (290)	1603 (305)	1729 (296)	1816 (337)	1632 (342)	1606 (411)	2204 (699)	2248 (609)
Dietary fat intake, E%	29.4 (4.6)	32.1 (4.9)	29.9 (4.8)	31.9 (5.3)	31.7 (5.0)	31.5 (6.0)	32.5 (6.2)	32.9 (6.4)
Dietary protein intake, E%	16.6 (2.8)	16.1 (2.5)	16.3 (2.6)	16.1 (2.4)	17.9 (3.2)	17.4 (3.9)	18.9 (3.5)	18.9 (3.6)
Dietary carbohydrate intake, E%	54.0 (4.7)	51.7 (5.2)	53.8 (5.3)	52.0 (5.5)	50.4 (5.6)	49.9 (6.3)	47.8 (6.4)	46.2 (6.8)
Physical activity, MET, h/wk	NA	NA	NA	NA	22.1 (22.1)	21.4 (18.4)	30.3 (26.2)	23.7 (22.9)
Smoking, %*	NA	NA	NA	NA	10.9	18.1	13.7	23.6

*Ctl*, control group; *I*, intervention group; *MET*, metabolic equivalent; *NA*, not available.

Data are mean (SD) or percentage.

* Those who smoke at least once a week.
